# Cytokine-Based Log-Scale Expansion of Functional Murine Dendritic Cells

**DOI:** 10.1371/journal.pone.0006674

**Published:** 2009-08-18

**Authors:** Yui Harada, Yasuji Ueda, Hiroaki Kinoh, Atsushi Komaru, Terumi Fuji-Ogawa, Aki Furuya, Akihiro Iida, Mamoru Hasegawa, Tomohiko Ichikawa, Yoshikazu Yonemitsu

**Affiliations:** 1 Department of Gene Therapy, Chiba University Graduate School of Medicine, Chiba, Japan; 2 Department of Urology, Chiba University Graduate School of Medicine, Chiba, Japan; 3 DNAVEC Corporation, Tsukuba, Ibaraki, Japan; 4 Department of Surgery and Science, Graduate School of Medical Sciences, Kyushu University, Fukuoka, Japan; New York University School of Medicine, United States of America

## Abstract

**Background:**

Limitations of the clinical efficacy of dendritic cell (DC)-based immunotherapy, as well as difficulties in their industrial production, are largely related to the limited number of autologous DCs from each patient. We here established a possible breakthrough, a simple and cytokine-based culture method to realize a log-scale order of functional murine DCs (>1,000-fold), which cells were used as a model before moving to human studies.

**Methodology/Principal Findings:**

Floating cultivation of lineage-negative hematopoietic progenitors from bone marrow in an optimized cytokine cocktail (FLT3-L, IL-3, IL-6, and SCF) led to a stable log-scale proliferation of these cells, and a subsequent differentiation study using IL-4/GM-CSF revealed that 3-weeks of expansion was optimal to produce CD11b^+^/CD11c^+^ DC-like cells. The expanded DCs had typical features of conventional myeloid DCs *in vitro* and *in vivo*, including identical efficacy as tumor vaccines.

**Conclusions/Significance:**

The concept of DC expansion should make a significant contribution to the progress of DC-based immunotherapy.

## Introduction

Dendritic cells (DCs) are unique antigen-presenting cells that can efficiently stimulate innate as well as acquired immune responses against pathogens and endogenous cancers. Over the last decade, there has been much anticipation about the potential for DC-based immunotherapy as a new therapeutic modality for cancers and infectious diseases; however, relatively limited efficacies have been reported in clinical studies [1]. Before DC-immunotherapy can be standardized, it will be necessary to resolve complex issues related to the overabundance of variables in the current clinical studies [2], including DC subtypes, antigen targeting *in vivo*, doses of DCs, and so on. Among these parameters, a possible critical issue underlying DC-immunotherapy is that only a limited numbers of DCs (roughly 10^6^ to 10^8^ DCs) are available from each patient in clinical studies, even via frequent aphereses for DC progenitor collection. Therefore, it is clear that the establishment of technology to expand the number of DCs would make the process of DC manipulation less invasive and would facilitate quality control in the industrial production of DCs. More importantly, experimental studies using dermal tumor and lung metastasis models have revealed that DC-based immunotherapy elicited a significant dose-response. The optimal dose for both models was at least 10^6^ DCs/30 g, roughly equivalent to 10^9^ DCs/patient according to the weight ratio (Kato T, Ueda Y, Yonemitsu Y, et al., unpublished data). Therefore, increasing the number of DCs would be expected to improve the efficacy of DC-based immunotherapy in a clinical setting. For these reasons, we here focused on the development of a technique for expanding functional murine DCs from bone marrow as a first step before moving to human materials.

## Results

Lineage-negative hematopoietic progenitors (HPs: CD45R^−^, CD5^−^, CD11b^−^, Gr-1^−^, TER119^−^, 7/4^−^) were prepared from bone marrow cells of female C3H/HeN as previously described [3], [4], and cultivated for 6 weeks under each cytokine (FLT3-L: FLT3-ligand; SCF: stem cell factor; IL-3: interleukin-3 or IL-6) that were previously shown to expand HPs [5]–[7], or their mixture (abbreviated as FS36) to assess their ability to expand the progenitor cells.

**Figure 1:**
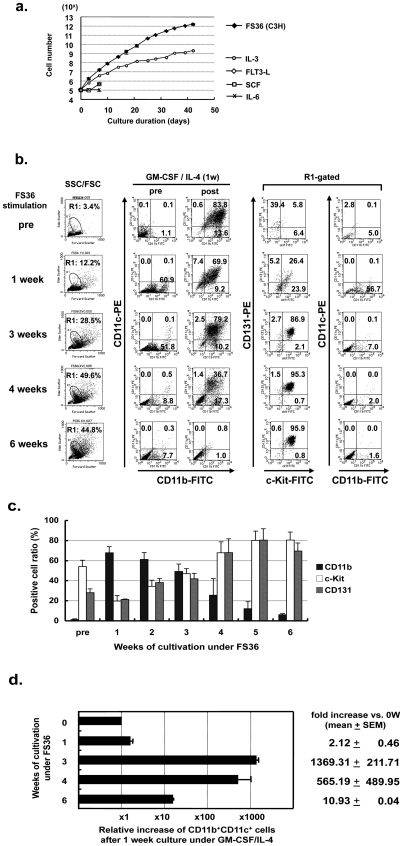
Cytokine-based expansion of bone marrow-derived murine DCs. Bone marrow (from female C3H/He)-derived lineage-negative cells (CD45R^−^, CD5^−^, CD11b^−^, Gr-1^−^, TER119^−^, and 7/4^−^) were enriched using a SpinSep mouse hematopoietic progenitor enrichment kit. These cells were subjected to progenitor expansion by various cytokines under a floating condition in an MPC treated flask. At each time point, the culture medium was replaced with new DC-differentiation medium (RPMI 1640 containing GM-CSF/IL-4) for 1 week. The data are expressed as the means+SEM. a. Growth curve of hematopoietic progenitor cells (HPs). Neither FLT3-L, SCF, nor IL-6 could stimulate the growth of HPs over 10 days. Only the use of IL-3 was associated with long-term growth, and this growth was greatly accelerated when a mixture of cytokines was used (FS36). Note the log scale on the HP cell number. b. FACS analyses indicating the time course of the scatter plot of HPs (left row), in populations of CD11c^+^CD11b^+^ cells at pre- and post-treatment with GM-CSF/IL-4 (middle two 2-center rows), and of the expression of c-Kit/CD131 (a receptor of GM-CSF/IL-3) and CD11c/CD11b in the R1-gated increasing population indicated in SSC/FSC (right two rows at right). These experiments were performed in triplicate, and showed similar results. c. Summary of triplicate FACS experiments representing the time course of positive cell ratios for CD11b, c-Kit, and CD131 of expanded HPs. Note the culture duration-dependent increase of c-Kit^+^ and CD131^+^ HPs. d. Bar graph indicating the relative increase of CD11b^+^CD11c^+^ DC-like cells produced after 1-week cultivation in GM-CSF/IL-4 using expanded HPs at various time points. The data was gathered from three independent experiments. Note that a period of 3 weeks was [optimal][most efficient], yielding a more than 3-log increase in the production of CD11b^+^CD11c^+^ DC-like cells.

Among these cytokines, only IL-3, but not FLT3-L, SCF, or IL-6, could expand HPs, a finding in agreement with those of previous reports [5], [6], over 10 days after floating cultivation in a low cell binding plate (Fig. 1a). The mixture of these cytokines, on the other hand, showed a more pronounced cell expansion; a nearly 2-log higher number of cells was found when FS36 was used, compared to that seen with IL-3, at 3 weeks. Finally, more than 10^12^ cells could be obtained from 10^5^ HPs ( = 10^7^-fold expansion) after 6 weeks of cultivation; therefore, we focused on the FS36 cytokine mixture. This experiment was repeated at least three times using female C3H/He mice, and representative results were also obtained using male C3H/He mice, female balb/c mice and C57BL6J mice (data not shown).

We next moved to a study assessing the yield of immature DCs from expanded progenitors. One-week cultivation under GM-CSF/IL-4 led HPs without expansion to CD11c^+^CD11b^+^ DCs efficiently, and similar findings were seen in the case of cells expanded for 1 or 3 weeks (Fig. 1b, middle 2 panels). However, the efficacy of the induction of CD11c^+^CD11b^+^ DC-like cells was dramatically decreased when HPs expanded for 4 weeks were used, with almost no such cells being obtained after 6 weeks of cultivation. Scatter plots demonstrated an increasing population of undefined cells which was gated by R1 during expansion (Fig. 1b, left panels); therefore, we characterized these cells by assessing the expression of surface markers. As shown in 2-panels in right of Fig. 1b and Fig. 1c, R1-gated cells were largely composed of c-KIT^+^/CD131^+^(a receptor for GM-CSF/IL-3) cells, suggesting more dedifferentiated hematopoietic cells [8] including granulo macrophage progenitors (GMP) [9], [10]; this hypothesis was supported by the result of an *in vitro* colony formation assay, which demonstrated increased numbers of CFU-c, but not formation of BFU-E (data not shown). We could obtained about 5,000 DCs from 100 HPs using conventional method. The yielded CD11c^+^CD11b^+^ DC-like cells was optimized by the use of HPs that had been expanded for 3 weeks, which resulted in a more than 1,000-fold increase in cells (approximately 7,000,000 CD11c^+^CD11b^+^ DC-like cells from 100 HPs) (Fig. 1d). Further FACS studies demonstrated that yielded CD11c^+^CD11b^+^ DC-like cells were negative for B220 (data not shown), suggesting that these cells were not plasmacytoid DCs.

**Figure 2:**
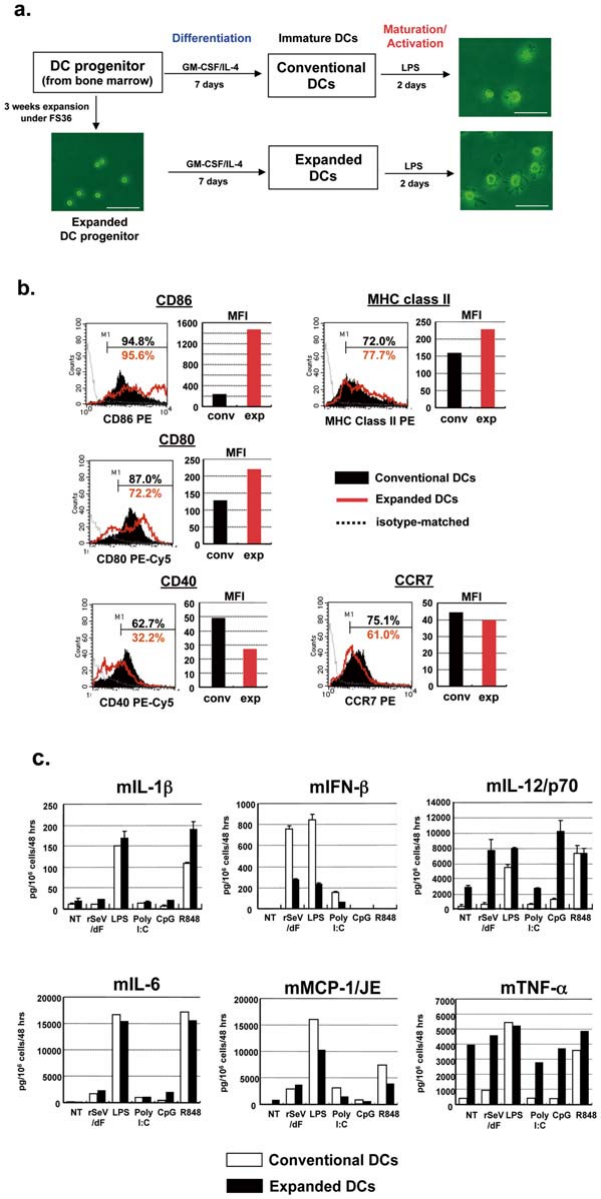
*In vitro* characterization of expanded murine DCs. a. Schematic diagram of expansion/differentiation/maturation/activation sequences and microscopic morphology of conventional and expanded DCs that were stimulated by LPS. Note that typical dendrites were found in both DCs. b. FACS analyses assessing the expression of typical surface markers. Conventional and expanded DCs after treatment with GM-CSF/IL-4 without further stimulus were subjected to FACS analyses. c. Expression of typical murine inflammatory cytokines/chemokines of conventional (open bars) and expanded (black bars) DCs in response to various stimuli for RIG-I helicase (rSeV) or Toll-like receptors (LPS for TLR-4, poly I:C for TLR-3, CpG-DNA for TLR-9, and R848 for TLR-7). The upper three panels (mIL-6, mIFN-β, and mIL-12/p70) were assessed by ELISA, and contain data from three independent experiments, and the bottom three panels (mIL-6, mMCP-1/JE, and mTNF-α) were performed using a Cytometric Bead Array (CBA) system and show one typical result taken from three independent experiments.

Next, we assessed whether CD11c^+^CD11b^+^ DC-like cells (expanded DCs) obtained from HPs after 3 weeks of expansion by FS36 might have typical features of myeloid DCs produced via a conventional culture method (conventional DCs) [3], [4]. As shown in Fig. 2a, both types of DCs demonstrated typical dendrites under a microscope at two days after culture in a medium containing lipopolysaccharide (LPS: 1 µg/ml). FACS analyses also showed the expression of typical surface markers of both DCs (CD40, CD80, CD86, MHC class II, and CCR7), although the expression level of each antigen varied between conventional DCs and expanded DCs (conventional DCs > expanded DCs: CD40, conventional DCs = expanded DCs: CCR7, and conventional DCs < expanded DCs: CD80, CD86 and MHC class II) (Fig. 2b). The expression of cytokine/chemokines in response to stimulators, including the F-gene deleted non-transmissible recombinant Sendai virus (SeV/dF) recognized by RIG-I [4], [11], LPS by Toll-like receptor 4 (TLR-4), poly I:C by TLR-3, CpG-DNA by TLR-9, and R848 by TLR-7, was commonly seen in both types of DCs (Fig. 2c). Among these, the responses of murine IL-12/p70 (mIL-12/p70) and mTNF-α to these stimulators were more pronounced in expanded DC-like cells compared to those seen in conventional DCs. These findings were basically identical to those seen using male C3H/He mice, female balb/c mice and C57BL6J mice (data not shown).

Next, we assessed the endo-/phagocytotic activity, a typical feature of antigen-presenting cells such as DCs, by FITC-dextran uptake assay. Repeated experiments demonstrated that both unstimulated immature DCs showed a similar uptake activity that was impaired by LPS but not by stimulation with rSeV/dF or poly I:C, and these findings were similar to those in our previous study [4]. The assay for the mixed leukocyte reaction (MLR) against allogenic antigen (C57BL6J) demonstrated an alloantigen-specific T-lymphocyte proliferation by both LPS-stimulated and unstimulated DCs, suggesting that both DCs enhanced alloantigen-specific T-cell proliferation.

**Figure 3:**
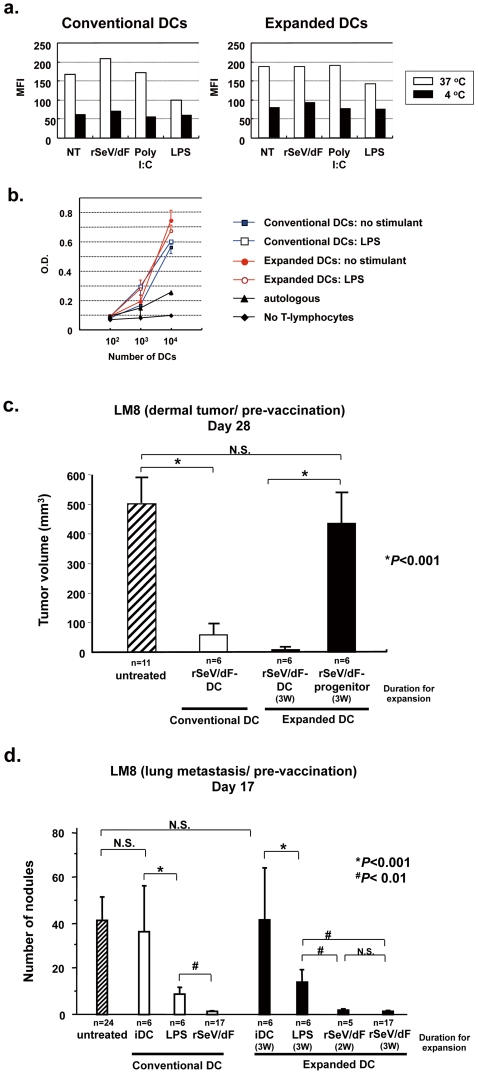
Assessment of functions that are typically seen in DCs. a. FITC-dextran uptake assay assessing endo-/phagocytotic activity, a typical feature of antigen-presenting cells such as DCs. This experiment was performed in triplicate, and showed similar results. b. A graph showing MLR activity for allo-antigen (C57BL6) by iDCs or activated DCs (C3H/He) by LPS derived from the conventional technique or expansion of HPs. c. Antitumor effect of subcutaneous vaccination with rSeV/dF-activated DCs that were derived from conventional or expansion techniques. Female C3H/He mice (7-week-old) were subcutaneously vaccinated via the left flank three times each weeks with 1×10^6^ conventional/expanded DCs pulsed with tumor lysate. Two days after the final vaccination, 1×10^6^ LM8 cells were inoculated intradermally into the left flank of mice. Note that 3-weeks expanded HPs treated with rSeV/dF did not show any effect on tumor growth. d. Antimetastatic activity via bolus intravenous injection of various DCs. Female C3H/He mice (7-week-old) were intravenously vaccinated with 1×10^6^ conventional/expanded DCs once via the tail vein, and 2 days later, 1×10^6^ of LM8 cells were inoculated intravenously. Seventeen days later, mice were sacrificed and the macroscopically recognized nodules on the surface of the bilateral lungs were counted.

The function of the immunostimulating activity of expanded DCs was further assessed using two different syngeneic mouse models of cancer vaccines *in vivo*—namely, subcutaneous inoculation forming dermal tumor (major effector: cytotoxic T-lymphocytes, or CTLs) and lung metastasis by intravenous injection (major effector: natural killer cells, or NK cells) using syngeneic (C3H/He) LM8 osteosarcoma [12]. We here used rSeV/dF as a DC-stimulator, because it has been shown that DCs treated by this modality demonstrated strongly enhanced antitumor immunity in dermal [3], [4] and metastatic [13] tumors. As shown in Fig. 3c, three-times weekly prevaccination through an intradermal route of conventional DCs treated with rSeV/dF significantly prevented tumor formation (*P*<0.001). Under the same treatment regimen, rSeV/dF-DCs that were obtained from HPs expanded for 3 weeks also showed a similar effect; however, rSeV/dF-treated and 3-weeks' expanded HPs without GM-CSF/IL-4 for DC differentiation did not show a significant antitumor effect. When conventional DCs were administered intravenously via the tail vein 2 days before tumor inoculation, DCs activated either by LPS or rSeV/dF but not immature DCs significantly prevented the lung metastasis; the effect was more pronounced when rSeV/dF-DCs were used, as shown in Fig. 3d. The representative findings shown in this figure were obtained in our previous study using rat prostatic cancer AT6.3 cells [13]. Similar results were also obtained in the present study using expanded DCs, even those obtained from HPs after 2 weeks of expansion.

Taken together, these findings led us to conclude that cells differentiated from expanded HPs could be considered functional and nearly equivalent to those obtained from the conventional method.

## Discussion

The growing demand for increased numbers of functional DCs, both for improved efficacy of DC-based immunotherapy and for use in the industrial production of DCs, has led us to develop an efficient method for the mass production of functional DCs. As an important first step, we established and optimized a two-step culture method, consisting of 3 weeks of expansion under FS36 followed by GM-CSF/IL-4, to expand murine DCs from bone marrow more than 1,000-fold, and we believe that this technique could be extended to human DCs. Furthermore, to the best of our knowledge, this is the first demonstration of an efficient, more than 3-log expansion of murine functional DCs *ex vivo*.

Basically, there are a considerable number of studies assessing the expansive ability of cytokines for hematopoietic series [5]; however, to our best knowledge, the first study attempting to expand murine DCs was published in 2000 by Feng et al [6] who used relatively complex cocktail of cytokines (GM-CSF, TNF-α, IL-7, IL-3, SCF, and FLT3-L) and single step cultivation. It is very difficult to make a direct comparison between this study and the present one, because no data are available regarding the basal number of DCs via GM-CSF/IL-4, they obtained more than 10^5^ DCs from 10^3^ of c-kit^low^ and c-kit^<low^ hematopoietic stem cells (HSCs). As usual, the conventional culture method using GM-CSF/IL-4 increases the final number of DCs from progenitors approximately 10-fold; therefore, the expansion efficacy in their report might be roughly estimated as ∼10-fold (in other words, 100-fold lower than those obtained by our regimen). Since these authors demonstrated that, among used cytokines, the combination of SCF and IL-3 but not FLT3-L did play a significant role in the DC expansion, findings apparently conflicting to the expansion of HPs suggesting the its critical role of FLT3-L [7]; therefore, it should be necessary to select cytokines appropriately selected for the target cell expansion.

We here demonstrated that expanded DCs showed similar characteristics, in views of morphology, surface markers, and biological functions, to those seen in conventional DCs. These findings, however, do not always indicate their equivalency at each developmental stage of a hematopoietic series. For example, repeated gene expression analyses using a microarray system have revealed the critical difference between conventional and expanded DCs on expression in some essential genes concerning hematopoietic differentiation was observed; i.e. *GATA-1*: conv. < exp. (6.3-fold), *GATA-3*: conv. < exp (12.0-fold), and inversely, *GATA-2*: conv. > exp (6.0-fold) (unpublished data). More importantly, it is not likely that FS36-expanded cells might be equivalent to monocyte-dendritic cell progenitor (MDP), which was highly enriched in Lin^−^/c-Kit^+^ as well as CD115^+^ (a receptor for M-CSF) [14], [15]; these expanded cells contained very small population of defined MDPs (less than 1 %, data not shown), even though the progenitor cells expanded by FS36 could be differentiated into DCs. Furthermore, our colony formation assay revealed that FS36-expanded cells couldn't form BFU-E (data not shown), suggesting that these cells may contain GMP rather than common myeloid progenitor (CMP). These findings suggest that cells showing typical and predefined features of DCs might be involved in relatively broad range of developmental stage of hematopoietic series. Even if this hypothesis is true, this would not undermine the value of developing a DC expansion system, because we believe that such a system would realize not only more efficient therapeutic outcomes but also mass industrial production.

This raises an important question: is the cytokine cocktail used in the current study applicable for the expansion of human DCs? To our knowledge, there is only one published study available to address this issue. This study used a sequential culture of CD34^+^ cells in medium containing FS36 followed by an alternative cocktail (FLT3-L, SCF, and trombopoietine) and showed a dramatic expansion (453-fold) in the cell number of progenitors; however, only ∼1×10^6^ DCs were produced from 1×10^5^ CD34^+^ cells [16]. We also confirmed this in our laboratory (unpublished), indicating that, unlike in the case of murine DCs, FS36 would not be the optimal combination for the expansion of human DCs. We have recently determined an optimal combination of cytokines, and which realized a very efficient expansion regimen (more than 10,000-fold) for human CD11b^+^/CD11c^+^/CD1a^−^/CD14^+^/MHC class II^+^/CD33^+^ DCs from CD34^+^ cells that are now under investigation for further characterization.

In summary, we here reported an optimized two-step culture method to generate highly expanded functional myeloid DCs from murine bone marrow. The concept and technology of log-scale expansion of functional DCs would significantly contribute not only to improved therapeutic efficacies but also to industrial mass production of functional autologous DCs for more efficient DC-based immunotherapy.

## Materials and Methods

### Ethics Statement

All animal experiments were performed in accordance with the approved protocols and advice of the Committee for Animals, Recombinant DNA, and Experiments Using Infectious Pathogens at Chiba University with respect to proper care and use of laboratory animals, and with the Law (No. 105) and Notification (No.6) of the Japanese Government.

### Mice, cell line, and rSeV/dF

Male and female C3H/He, C57/BL6, and balb/c mice (7 weeks old) were purchased from Shizuoka Laboratory Animal Center (Hamamatsu, Shizuoka, Japan) and kept under specific pathogen-free conditions. The murine osteosarcoma cell line, LM8, was purchased from RIKEN BioResource center (Tsukuba, Ibaraki, Japan). The preparation, recovery, titration, and storage of F-defective and non-transmissible recombinant SeV used in this study (SeV/dF) were performed as previously described [4], [17]. The virus yields are expressed in cell infectious units (CIU) [17], [18].

### Generation of expanded DCs

Conventional DCs were obtained from mouse bone marrow precursors as described previously with minor modification [3], [4]. Briefly, bone marrow cells were harvested from femurs and tibias, and lineage antigen-positive (CD45R, CD5, CD11b, Gr-1, TER119, 7/4) cells were removed using a SpinSep mouse hematopoietic progenitor enrichment kit (StemCell Technologies). For expansion, these lineage-negative cells were cultured in 20 ng/ml murine FLT3-L, 10 ng/ml murine SCF, 10 ng/ml murine IL-3 and 10 ng/ml murine IL-6 (FS36) in RPMI 1640 medium. The culture was started at 5−10×10^4^ cells/ml and serially passaged at 2.5×10^5^ cells/ml every 3 or 4 days. During culture, cells were not exceeded 1.5×10^6^ cells/ml. Subsequently, expanded cells were cultured under 20 ng/ml murine GM-CSF (Peprotech, London, UK) and 20 ng/ml murine IL-4 (Peprotech) in RPMI 1640 medium. On day 4, the cultures were refreshed by adding a half volume of culture medium supplemented with GM-CSF and IL-4 at the same concentrations. On day 7, DCs were collected and seeded at 1×10^6^ cells/ml and then incubated with stimulators. Cells were cultured on an MPC treatment 6-well plate (MD6 WITH LID LOW-CELL-BINDING; Nalge Nunc International K.K., Tokyo, Japan).

### Flow cytometric analysis

Two days after stimulation, cells (1×10^5^) were stained with the following FITC-, PE-, or PE-Cy5-conjugated monoclonal antibodies (mAbs): CD11b, CD11c, CD40, CD54 (ICAM-1), CD80, CD83, CD86, CD197 (CCR7), MHC Class II (eBioscience), and CD11c (Pharmingen, San Diego, CA). The appropriate conjugated isotype-matched IgGs were used as controls. Cells were analyzed using a FACScalibur with CellQuest software (Becton Dickinson, Tokyo, Japan).

### Fluorescein isothiocyanate (FITC)-dextran uptake

Cells were suspended in RPMI 1640 with 10% FBS and incubated with 1 mg/ml of FITC-dextran (M.W. = 40,000; Sigma-Aldrich, Tokyo, Japan) for 30 min under separate conditions, at 4°C and 37°C. Cells were washed with ice-cold phosphate-buffered saline (PBS) and labeled on ice with a PE-conjugated mAb for CD11c. The CD11c-positive mean fluorescent intensity (MFI) of FITC was analyzed by FACScalibur. The uptake was measured at 48 h after stimulation, and was calculated as the change in MFI between cell samples incubated at 37°C and 4°C.

### Allogeneic mixed lymphocyte reactions (allo-MLRs)

Spleen cells were obtained from allogeneic C57/BL6 mice. After lysing red blood cells using VersaLyse Lysing Solution (BECKMAN COULTER, Tokyo, JAPAN), lineage antigen-positive (CD11b, CD19, CD45R, CD49b, TER119) cells were removed by using the SpinSep mouse CD3^+^ T cell enrichment kit (StemCell Technologies), and used as responder cells. In vitro-generated immature conventional/expanded DCs, as well as rSeV/dF-DCs, LPS-DCs, and Poly (I:C)-DCs stimulated on day 7, were collected at day 9. These DCs were treated with 20 ug/ml MitomycinC (MMC) for 1 h at 37°C and then used as stimulator cells. Allogeneic responder cells (1×10^5^ cells/wells) were cultured in triplicate in a 96-well round-bottom microplate with different numbers of stimulator APCs (the APC-to-T cell ratios were 1∶10, 1∶100, and 1∶1000). Cultures were maintained in a humidified atmosphere at 37°C and 5% CO_2_. The thymidine analogue BrdU was added on day 4 followed by quantitation of incorporated BrdU after a further 2 h of culture using an ELISA-based cell proliferation kit (BrdU colorimetric, 1647229; Roche, Mannheim, Germany) according to the manufacturer's protocol.

### Cytokine assay

#### ELISA

The conventional/expanded DCs were cultured with rSeV/dF (MOI = 50), LPS (1 ug/ml), or Poly (I:C) respectively for 48 h, the RPMI1640 medium (1×10^5^ cells/ml) was refreshed, and DCs were incubated for 24 h. The culture media were subjected to the concentration of murine IL-1β, IFN-β, and IL-12p70 by quantitative sandwich enzyme immunosorbent assay using a mouse specific ELISA kit (Biosource, Camarillo, CA) according to the manufacturer's instructions.

#### Cytokine bead array

The same media were subjected to the concentration of mouse IL-6, MCP-1, TNF-α by a Cytometric Bead Array (CBA) Mouse Inflammation Kit (BD Biosciences, San Diego, CA) using a FACScalibur and CellQuest software (Becton Dickinson, Tokyo, Japan).

### Vaccination

#### Skin tumor

Mice were subcutaneously vaccinated via the left flank three times each week with 1×10^6^ of conventional/expanded DCs pulsed with tumor lysate. Two days after the final vaccination, 1×10^6^ LM8 cells were inoculated s.c. into the left flank of mice. The tumor volume was assessed using microcalipers every 3 or 4 days after the inoculation of LM8 cells according to the formula: tumor volume (mm^3^) = S^2^×L/2, where L and S indicate the size in millimeters of the longest and shortest part, respectively.

#### Lung metastasis

Mice were intravenously vaccinated once with 1×10^6^ conventional/expanded DCs pulsed with tumor lysate *ex vivo*. Two days after the vaccination, 1×10^6^ LM8 cells were inoculated intravenously via the tail vein. Seventeen days after the injection of LM8 cells, the mice were sacrificed and the metastasis was quantified according to the number of metastatic nodules on the surface of the lung.

### Statistical analysis

All data were expressed as the means+SEM, and were evaluated statistically by one-way ANOVA or Mann-Whitney *U*-test when appropriate, respectively. The statistical significance of differences between groups was determined using the Scheffe's test, and values of P<0.05 were considered statistically significant.
